# Orbital Prosthetic Rehabilitation in “ADAM Complex” Multiple Orofacial-Cleft Disruption Syndrome

**DOI:** 10.1155/2013/809479

**Published:** 2013-05-12

**Authors:** Aparna Barabde, Shailesh M. Barabde, Ashish Bhagat, Amar Thakare

**Affiliations:** ^1^Department of Prosthetic Dentistry, V.Y.W.S. Dental College & Hospital, Amravati 444 602, India; ^2^Dr Barabde Dental Clinic, Badnera Road, Rajkamal Square, Amravati 444 601, India; ^3^Department of Obstetrics & Gynaecology, P.D.M. Medical College, Amravati 444 602, India

## Abstract

To be human is great; to look human is wonderful! It is nature's greatest gift! Mother nature's womb is the safest place on earth for any life, but the calamity strikes and no one knows how Hence, Treasure your exceptions!, since nature seems nowhere accustomed more openly to display, its secret mysteries than in cases where it shows traces of its workings apart from the beaten path. A dismorphological pattern of congenital oro-craniofacial and limb defects which is a rare form of amniotic rupture sequence required persistent coordinated efforts of multiple disciplines and had manifested as bizarre orofacial clefting, cat eye syndrome with an ectopic eye, and aberrant tissue band lesions on limb. The challenge was to meet the child's clamour for functional demands on premature exposure to open world and was overcome through a phased treatment implementation. Anophthalmos resulting from multiple ophthalmic surgeries for aberrant ectopic left eye and cat eye syndrome of right eye required a staged sequential preemptive planning for a successful outcome. Every phase of fabrication of orbital prosthesis comes with an impending challenge. Thus, a well-defined technique eliminating the common errors and creating a natural looking prosthesis, in the face of limitations, is imperative.

## 1. Introduction

The naturally vibrant and dynamically variable region of the orbit poses innumerable challenges in making an indiscernible replica of the opposing eye. Simulation of nature through rehabilitation forms the primary goal for state-of-art restoration in these genres of maxillofacial prosthodontic modelling. 

Dr. Leisely, Sweden, 1991 has said no matter how accurate your diagnosis is, no matter how effective your prognosis; the entire success in disease management depends upon the patient's will to come out of the odd.

Thus, to create the perfect esthetic illusion of normal eye poses multiple challenges as we are attempting to restore a moving organ with a static prosthesis. An interdisciplinary case report provides the perfect medium for determining plausible solutions to overcome the challenges posed at every step of the wall till we achieve a successful outcome, thus, turning a distressing scenario of nightmare into a metaphysical reality.

## 2. Clinical Features

 “Adam complex” disruption syndrome [1] is a rare, complex, fetal, fatal spectrum of malformations portraying as dismorphological pattern of congenital orofacial and limb defects involving all three basic types of anomalies, that is, disruptions, deformations, and malformations, whereas the mnemonic term “ADAM” complex stands for A: Amnion, D: Deformity, A: Adhesion, and M: Mutilation.

**Figure 1:**
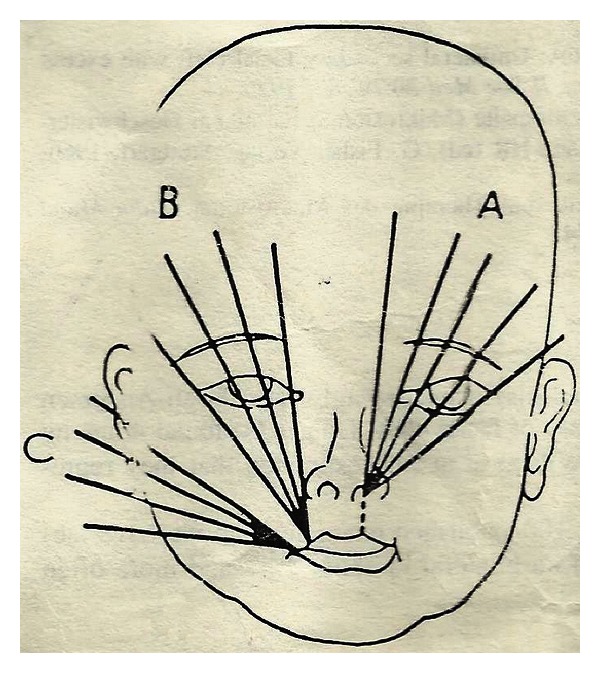
Oblique facial clefts.

**Figure 2:**
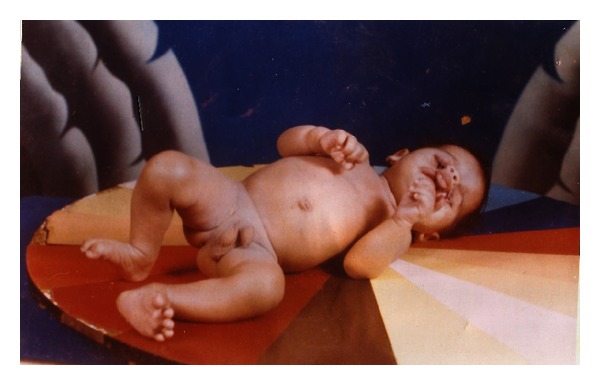
Gaurav at birth.

**Figure 3:**
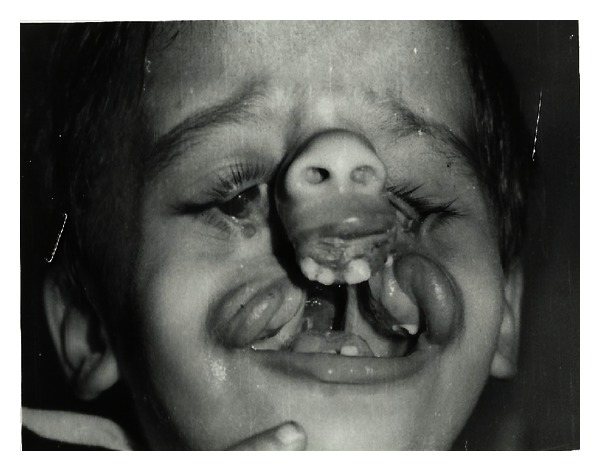
ADAM-complex features.

The clinical features include:

1. craniofacial defect-facial clefts CL(L) and (P), asymmetric microphthalmia (Figures 1, 2, and 3),
2. visceral defect—gastroschisis, omphalocele,
3. limb defect—constriction ring or amputations (Figure 2).

*Epidemiology*. 1 : 1,200–1 : 15,000 live births, extremely rare congenital abnormality. Also known as Amniotic band syndrome [2].

**Figure 4:**
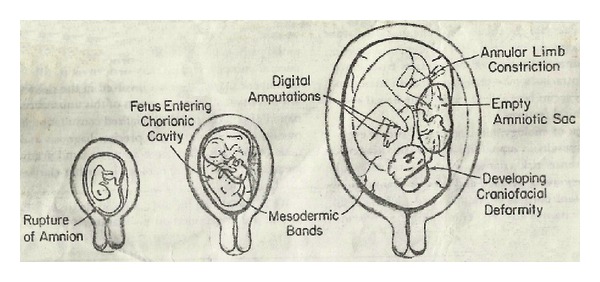
Torpin's exogenous theory.

*Etiology*. Remains unclear. Factors postulated are based upon Streeter's endogenous [3] and Torpin's exogenous theory [4]. Early amnion rupture due to genetic, toxic, congenital, endocranial factors may determine the process (Figure 4).

*Antenatal Diagnosis*. Second trimester of pregnancy by elevated amniotic *α*-fetoprotein levels, or specialised, genetic consultation, ultrasound.

*Differential Diagnosis*. One needs to exclude similar conditions like Meckel's syndrome, Frontonasal dysplasia, and so forth, to confirm the diagnosis.

A remarkable feature is that the general intelligence is normal.

Due to complexity of this association a complete study and adequate treatment requires multidisciplinary collaboration. Thus defects in oral and craniofacial tissues, resulting from congenital abnormalities present a formidable challenge. At birth surgical removal of ectopic left eye leads to Anophthalmos whereas respiratory distress required prompt resuscitation.

## 3. Clinical Report 

A 7-year-old male child “Gaurav” reported to the Department of Prosthodontics with chief complaint of missing left eye and inadequate vision of right eye. He is the first child of a healthy nonconsanguineous parents with no family history of any congenital defect. His mother gave history of bacterial infection and he was born prematurely in 30th week of gestation. At birth surgical removal of ectopic left eye and prompt resuscitation for respiratory distress were required. 

 Medical and dental history revealed that he is operated for ectopic left eye. He has undergone repeated surgeries (8 times) for correction of intraoral, extraoral, and limb defect. 

**Figure 5:**
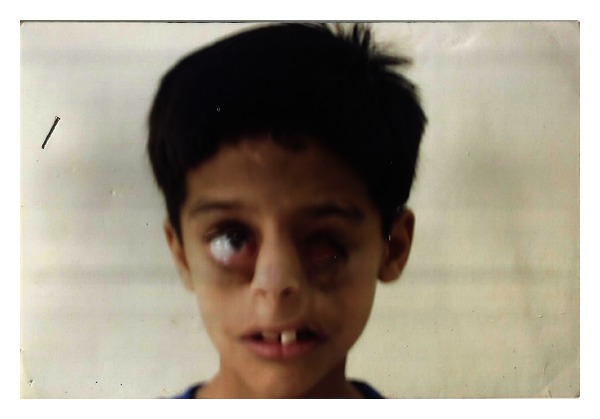
Frontal view showing ocular defect.

Examination revealed Anophthalmos with left eye, leading to symptoms of postenucleation socket syndrome like superior sulcus deepening and lower lid laxity; coloboma of right eye with multiple scar marks and variable degree of cleft fragment approximation along with surgically repaired bilateral oblique facial cleft, bilateral maxillary cleft lip (and palate), and floating premaxilla is seen. An extensive pigmentation on right forearm for constriction band lesion correction is evident (Figure 5).

Oroocular cleft type I (medial to infraorbital formen, according to Boo Chai's classification) and cleft number 4 (Tessier classification) mixed group for all facial clefts was determined.

**Figure 6:**
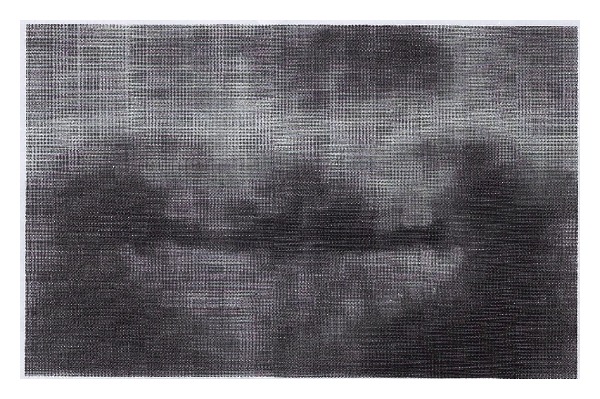
Postsurgical OPG view.

Postsurgical radiograph showed that both the maxillary antrum were hypoplastic but not cleft and there was funnelling of orbital floor (Figure 6).

Intraoral examination revealed bilateral fibrous band from lips to canine area to palate and proclinated anterior teeth causing typical gold fish appearance.

The most miraculous learning occurs when an infant turns into an eloquent child and no one knows how! It is by imitation at its pristine best! At 7 years of age, apart of missing eye Gaurav's chief complaint was flat cheeks, drooping eye lids, and cleft alveolar ridge and palate.

“He had but one eye; but the pocket of prejudice lies in favour of two.” The crippling effect of the defect on the growing child's mind cannot be overemphasized but this positive attitude was evident.

## 4. History and Review of Literature

Although interest in congenital malformations dates back to antiquity, a recognizable pattern of anomalies associated with oblique facial cleft was described as early as in 18th century in Latin—by Von Kolmus (1732) [1] known as father of dysmorphology and later by Walter Dick (1837) [1].

George Bartisch [5] in 1583 first described removal of the orbital contents.

Artificial eyes, ears, and noses have been found in Egyptian mummies (Popp 1939).

 Ambroise pare (1560) pioneer in maxillofacial prosthesis suggested use of glass and porcelain eyes for the first time.

Pierre Fauchard (1728) father of modern dentistry used prosthesis for a French soldier whose face was mutilated in war and called as “Gunner with as silver mask.”

Until World War II, the glass eye was most popular eye manufactured but was difficult to manufacture and hazardous and cannot be modified.

M. U. Ludwig (1835) fabricated eyes for dolls later used for patients.

Muller Uri in 19th century suggested vulcanite, cellulose and glass eyes. 

Sykes [6] in 1944 used acrylic physiologic ocular prosthesis.

Niiranen (1947) suggested modified acrylic eyes; K. E. Brown advocated characterization with rayon fibres. James et al. [7] (1976) suggested Graphic ocular locator. Chalian et al. [8] mentioned hollowup of prosthesis. Taicher et al. [9] approved of modified stock eyes and prefabricated eye shell customization. R. S. Schmerber (1986) regarding Iowa wax for modified impression for ocular prosthesis; are some of pioneers for suggested procedure [10].

### 4.1. Recent Improvements in Art of Ocular Rehabilitation [11]

1. Garonzick (US patent number 6530953) used method of magnetically coupling prosthesis with ocular implant.
2. Self-lubricating ocular prosthesis (dispensing button on demand).
3. Ocular prosthesis to simulate human pupil dilation.
4. Use of a light cure urethane dimethacrylate to minimize allergy.
5. Digitization by scanning the eye socket, digital impression, iris photographs, and discs deposited on posterior scleral portion and joint to anterior scleral portion.
6. CAD/CAM software to generate geometric models.
7. Use of photo editing software for change in colour, size, diameter, and multiple depth layer correction of eye.
8. Digital model with milling flanges and exported STL files.
9. Motility implant and use of ball and sphere in implants.
10. Bioeye, hydroxyapatite coralline ocular implant (conceived by Arthur C. Pellry, 1980).

## 5. Treatment Goals for Ocular Sclupture

The ocular prosthesis is aimed to provide the following benefits:

1. life like appearance and function,
2. protection and preservation of tissues,
3. therapeutic and healing effect,
4. intimate adaptation, equal distribution of pressure to tissue surface, massaging effect, and lubricating effect,
5. noninvasive procedure,
6. functional ocular movement, retention, and lid support,
7. adjustability to customize, reline, or remake to adapt to future growth and development changes,
8. enhanced natural feel, acceptability and moral upliftment,
9. comfortable and economical,
10. restore balance of face.

### 5.1. Ocular Sculpture Methodology

“To extract/remove is human but to restore is Divine!”

Rehabilitation is defined as the combined and coordinated use of medical, social, educational and vocational measure for training and retraining the individual to the highest possible level of functional activity (Park) [12].

The state-of-the art clinical methods and proposed benefits of ocular rehabilitation have changed significantly from those described by the early proponents of the technique.

Conventional processes that are currently in use for manufacturing an ocular prosthesis have been used for more than 6 years. The esthetic and functional outcome of the prosthesis is superior to the stock ocular prosthesis. They traditionally begin with the preparation of an impression of the anophthalmic eye socket. A confining impression tray is selected and placed into the socket anterior to the globe and posterior to the eyelids.

Unfortunately, following multiple ophthalmic invasive surgeries, lack of postoperative socket care led to constriction of the eye socket.

**Figure 7:**
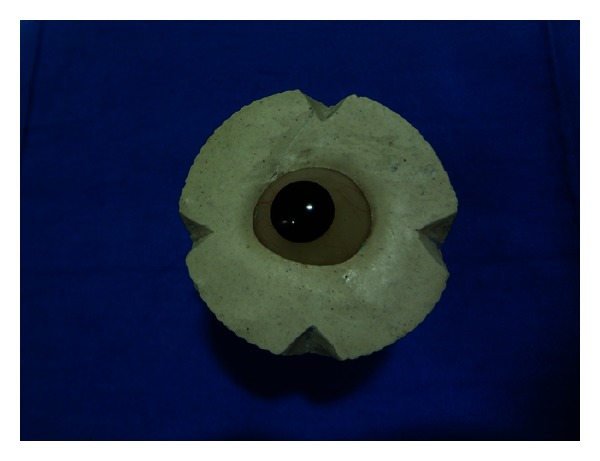
Invested eye shell.

**Figure 8:**
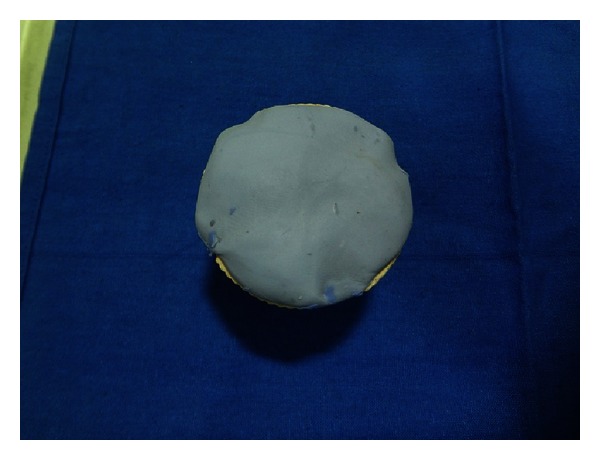
PVS cope preparation.

(1) Surgical dipping procedure of eye socket is carried out and after six weeks of healing period an impression for ocular conformer is made. In order to maintain the orbit volume an acrylic conformer is placed to form the cul-de-sac for lid pocket to hold the eye in place.

(2) *Ocular tray fabrication*. To fabricate an ocular tray to make an impression of eye socket, a prefabricated stock eye that optimally fits the socket is selected. The tissue surface of the eye is lubricated and invested in dental plaster to the height of contour. After plaster was set notches are made on its edges. Polyvinyl siloxane putty (Reprosil, Type I Dentsply, Germany) is mixed and adapted over the top of invested prosthesis into notched edges (Figures 7 and 8). After putty was set, it was removed and a bevelled sprue hole is made in the centre of the polyvinyl siloxane cope.

**Figure 9:**
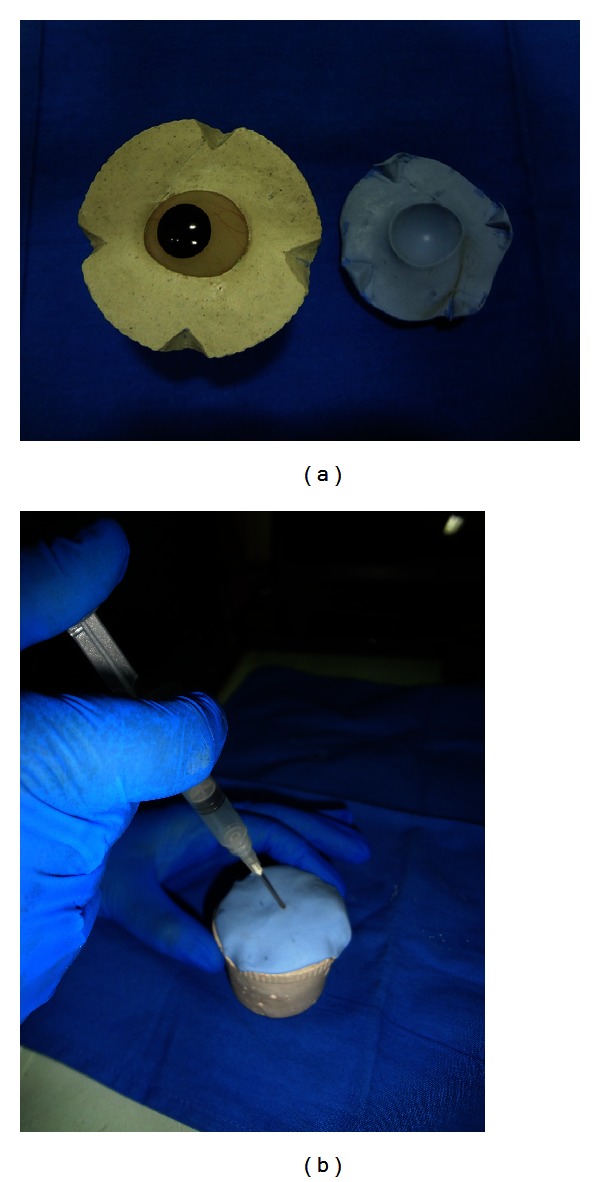
Fabrication of clear acrylic ocular tray.

The eye shell is then removed from the mould, plaster surface is lubricated and putty cope is replaced on the mould. The mould is filled with self-cure acrylic resin (DPI RR Cold Cure Bombay Burma trading Corp. Ltd.) mix (Figure 9). Thus, formed clear acrylic ocular tray is removed from the mould. Multiple perforations are made, polished, trimmed, and disinfected. It is tried in patient for overextension and orientation.

**Figure 10:**
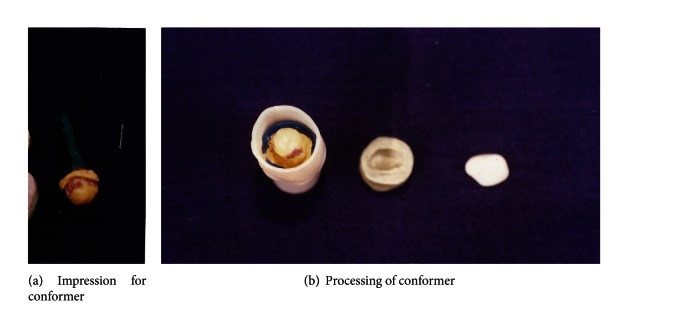
Fabrication of conformer.

(3) *Fabrication of conformer*. The tray is marked for iris and sclera portion for better handling. An acrylic stick is attached to the iris (centre) to act as a handle to facilitate removal of the ocular tray. Appropriate increments by low fusing impression compound were added to restore normal opening and to provide support to iris. The patient was asked to move his natural eye in various directions till material had set to obtain a functional impression. Subsequently a wash impression was made in light bodied elastomeric impression material. The entire assembly is flasked and the conformer prosthesis was processed in heat cure clear acrylic resin (DPI RR Heat Cure Bombay Burma trading Corp. Ltd.) and was polished, disinfected, and checked for fit. The conformer is retained in all times for 8 weeks therapeutic period to maintain socket volume (Figure 10).

(4) *Selection and orientation of eye shell*. A stock eye is selected that matches the colour and size of the sclera and iris-pupil complex of contralateral natural eye. It was modified by trimming and staining to match and resemble natural eye and rechecked for precise fit to the socket.

Ocular prosthesis was positioned to simulate the right eye with a reference mark placed at the midline and a Boley's gauge [8] was used to verify and confirm the mediolateral placing.

**Figure 11:**
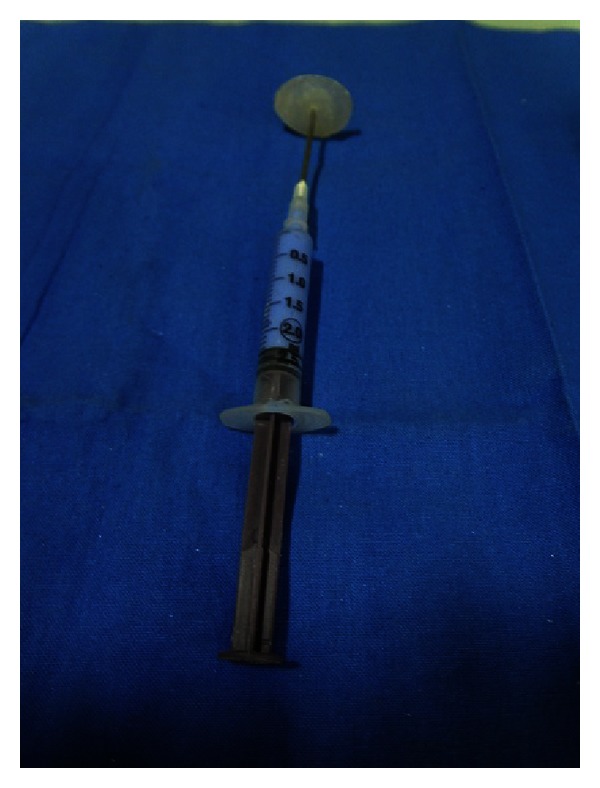
Ocular impression tray.

 (5) *Fabrication of acrylic portion of eye prosthesis*. First, a conforming impression tray is prepared as before using the selected eye and placed into the socket anterior to the globe and posterior to the eyelid. A syringe with needle (shortened length 10 mm) is secured to ocular tray with cold cure acrylic resin (Figure 11). The syringe is loaded with low viscosity poly-vinyl siloxane (Reprosil, type I, Dentsply, Germany).

**Figure 12:**
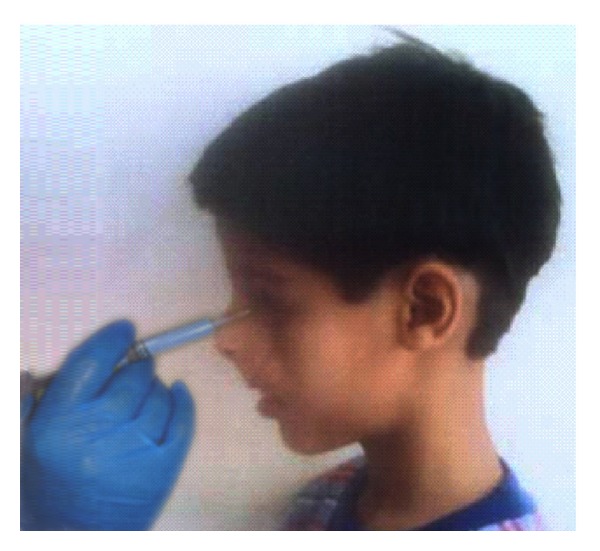
Impression for ocular prosthesis.

An impression material is then introduced into the eye socket via a tube protruding from the anterior surface of the impression tray and projecting out between the lids by means of a syringe connected to the tube (Figure 12).

The impression material is injected with the tray into the eye socket, patient is instructed to perform all movement of eye to maintain orientation, opening and closing, and support to lids. After removal of impression material stock eye shell replaces the ocular tray and rechecked in eye socket.

**Figure 13:**
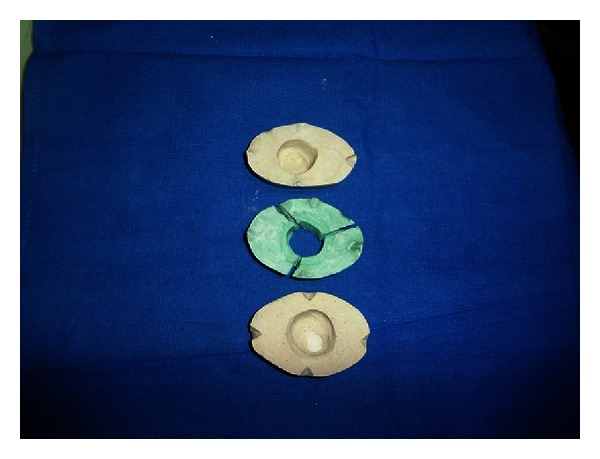
Three-piece dental stone mould (middle piece intentionlly fractured).

The complete assembly together is first invested with dental plaster till the height of contour and then with the stone (Tpye-3, Goldstone Asian Chemicals) and dental plaster to get a three-piece mould. A rounded acrylic elevation is attached at the junction of sclera and iris portion of the eye to allow reorientation of the shell during processing. (Figure 13). Thus obtained ocular prosthesis is then customized.

 (6) *Orbital prosthetic sculpture and customization*. A few veins are added to the sclera to improve the match. Silk fibres are added to duplicate the veining patterns of the contralateral eye with a thin layer of tooth coloured resin to hold. The prosthesis is then placed in a drying oven to prepare it for placement of clear acrylic resin over the anterior surface.

**Figure 14:**
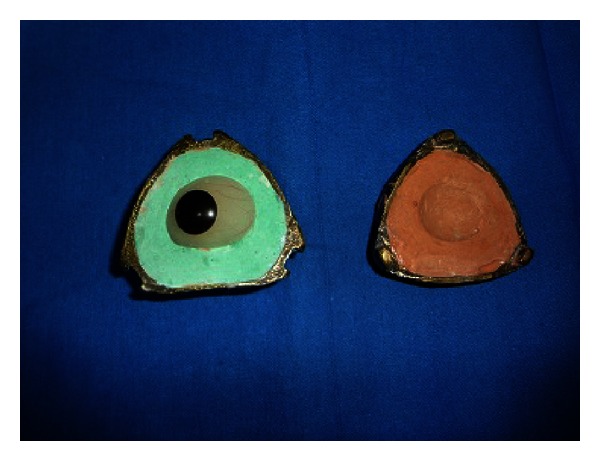
Processing for customized ocular prosthesis.

The bulge of eye and additional wax carving is gained as of contralateral eye by addition of wax (Wax number 2, Dental products, A.P.) onto tissue surface. The tissue surface of the prosthesis is again relined with soft tissue conditioner (Coe-soft GC America, Inc.) and placed into the socket to record functional ocular movement of eye; thus, retained tissue site of ocular prosthesis is invested, dewaxed, and packed with heat cure acrylic resin (DPI RR HC Bombay Burma trading Corp. Ltd.) (Figure 14).

The final assessment of position of eye, anatomic contour, and shaping is carried out. 

The entire assembly is cured in 70°C water bath for one and half hour, followed by 100°C water bath for 20 minutes. After deflasking it is polished thoroughly and is disinfected in 0.5% chlorhexidine and 70% isopropyl alcohol for 5 minutes and rinsed in sterile saline solution and inserted.

**Figure 15:**
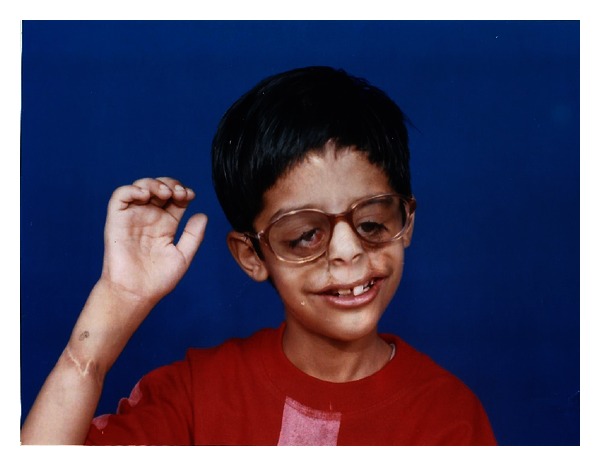
Child with customized ocular prosthesis in position.

Thus, customized ocular prosthesis is based on patient's existing socket volume and anatomy and conformed accurately to socket with intimate adaptation to internal tissue surface of socket hence increases the movement of the prosthesis and enhances its natural appearance (Figure 15).

TLC principles in child management: parental counselling and education regarding nature, functioning and limitations of orbital prosthesis, care, maintenance protocol, nutritional therapy and also awareness for importance of periodic checkups were made before start of the treatment. The prior laid down goals of sculpture are thus achieved.

## 6. Discussion

The importance of an orbital prosthesis with acceptable esthetics and reasonable motility in restoring the natural appearance in patients with anophthalmia is recognized since long [13].

Beumer et al. [14] state that a prefabricated resin eye should not be used because of lack of intimate contact. 

This is true if a prefabricated eye shell is selected and ground to close fit. 

However, when prosthesis is customized to the patient using proper impression technique, distribution of pressure and intimate adaptation of the modified prosthesis to the tissue surface of the defect provide movement to and aesthetics to rehabilitation. Traditionally chosen customization of prefabricated acrylic eye shell (Taicher et al. [9]) method is based expounding on the virtues of clinical experience.

Organic insult, loss of function, and appearance have serious harmful effects on the total adjustment of the patient to his life situation.

 Early treatment is important not only due to esthetic and functional concern but also for the positive psychological impact it has on the child. The treatment planning in this deformity is dependent upon the age, socio-economic status, type and severity of defect and the intra-oral situation at the time the treatment is planned. In recent times there has been a paradigm shift to enhance outcome by moulding for the growth and development than the surgery alone.

The challenge was to meet the infants clamour for functional demands on premature exposure to open world and was overcome through phased treatment implementation. Prosthodontic protocol involved expedient feeder prosthesis at birth with presurgical orofacial orthopaedics (PSIO), nasoalveolar moulding (PNAM), and maxillary expansion prosthesis apart from orbital prosthesis. Early PNAM represents a paradigm shift from traditional methods of PSIO, whereas surgical protocol involves removal of ectopic eye, coloboma of right eye, limb corrections, and craniofacial reconstructive correction for facial clefts, floating premaxilla and nasal deformities.

The components of ongoing treatment include future consideration for an artificial eye implant placement. The astuded guidance by obstetrician, paediatric plastic surgeon, syndromologist, speech therapist, and child psychotherapist was mandatory.

After initial surgeries inadequate parental counselling returned the child and was lost to followup for not less than 7 years of age. Final justification of these creations depends on the quality of life they prolong.

Thus it would be only when multiple disciplines together called forth and regard devotional care can we merit the most profound reverence towards life like restorations.

## 7. Conclusion

A well-made properly planned and functionally molded stock eye prosthesis maintains its orientation as patient performs various eye movements. Defects in orbitocraniofacial tissues resulting from congenital origin present a formidable challenge and restoration of these tissues is a subject of clinical, basic science, and engineering concern. Hence, primary goal of team work is not only to remove and prepare early replacement for diseased organ but to permit the person rather than the organ perse to function in a relatively normal manner. 

Orbital sculpture with digital concept and motility implants is emerging rapidly on the hi-tech horizon. Also prosthesis to simulate human pupil dilation is no longer a distant dream. Hence, this field of dentistry knows no bounds; it crosses the realms of oral cavity, to the extraoral structure and ventures beyond the horizon to restore other facial structures including eyes creating the so-called “healthy beauty”.

“The eyes are the windows of the soul.” The spark in Gaurav's eyes truly reflects his envisioned soul. This in turn is our inspiration and the golden reward.
